# Comparison of ultrasound guidance with landmark guidance for symptomatic benefits in knee, hip and hand osteoarthritis: Systematic review and meta‐analysis of randomised controlled trials

**DOI:** 10.1002/ajum.12386

**Published:** 2024-04-19

**Authors:** Win Min Oo, James Linklater, Md Abu Bakar Siddiq, Kai Fu, David J. Hunter

**Affiliations:** ^1^ Department of Physical Medicine and Rehabilitation, Mandalay General Hospital University of Medicine Mandalay Myanmar; ^2^ Rheumatology Department, Royal North Shore Hospital, and Sydney Musculoskeletal Health, Kolling Institute, Faculty of Medicine and Health The University of Sydney Sydney New South Wales Australia; ^3^ Castlereagh Imaging Centre Sydney New South Wales Australia; ^4^ Department of Orthopedic Surgery Shanghai Sixth People's Hospital Affiliated to Shanghai Jiao Tong University School of Medicine Shanghai China

**Keywords:** intra‐articular injection, landmark injection, osteoarthritis, ultrasonography, ultrasound

## Abstract

**Introduction:**

More than half of the patients with moderate and severe osteoarthritis (OA) report unsatisfactory pain relief, requiring consideration of intra‐articular (IA) injections as the second‐line management. Ultrasound‐guided IA injection has proven evidence of higher accuracy in administering IA injectates into the joints than landmark‐guided or blind IA injections. However, questions remain about translating higher accuracy rates of ultrasound‐guided injection into better clinical improvements. Therefore, we examined the symptomatic benefits (pain, function and patient satisfaction) of ultrasound‐guided injection in knee, hip and hand OA compared with blind injections by synthesising a systematic review and meta‐analysis of randomised controlled trials (RCT).

**Methods:**

PubMed, Medline and Embase databases were searched for eligible studies from their inception to August 28, 2023.

**Results:**

Out of 295 records, our meta‐analysis included four RCTs (338 patients with knee OA), demonstrating significant improvement in procedural pain [−0.89 (95% CI −1.25, −0.53)], pain at follow‐up [−0.51 (95% CI −0.98, −0.04)] and function [1.30 (95% CI 0.86, 1.73)], favouring ultrasound guidance. One single study showed higher patient satisfaction with ultrasound guidance.

**Conclusion:**

Ultrasound‐guided IA injection provided superior clinical outcomes compared with landmark‐guided IA injection.

## Introduction

Osteoarthritis (OA) is a chronic joint disorder prevalent in ageing populations, mostly affecting the knee (61%), hand (24%), hip joints (5%) and other synovial joints (10%).^1^ No drugs have yet approved to modify the structural manifestations of OA.^2^ Current OA management options include (i) non‐pharmacological management such as weight reduction, lifestyle changes and exercises, (ii) pharmacological options such as paracetamol, non‐steroidal anti‐inflammatory drugs (NSAIDs) and intra‐articular (IA) therapies such as steroids and hyaluronic acids and (iii) surgical interventions which are typically reserved only for end‐stage OA, as a last resort.^3^ However, long‐term use of commonly used analgesics is not recommended due to adverse effects related to the gastrointestinal, cardiac or renal systems.^4^ In a recent, real‐world, study of OA patients with moderate to severe pain (n = 29,886), 56.1% reported unsatisfactory pain relief from analgesics due to contraindication, intolerance or failed NSAID therapy.^5^ Under such circumstances, most guidelines of major professional societies recommend the use of IA therapies as the second line of treatment as part of conservative management.^6^ Local administration of drugs into the OA joints has the advantage of increased bioavailability and high efficiency while limiting the systemic exposure and off‐target effects.^7^

Traditionally, since 1951, drugs have been administered into the knee joint blindly, solely using physical examination and guidance from anatomical landmarks (landmark‐guided injection).^8^ However, there are difficulties and inaccuracies in injecting into the joint and risks for damage to surrounding structures, especially in OA joints due to bony spurs and deformities.^9^ On the contrary, several systemic reviews showed that ultrasound‐guided IA injection showed greater accuracy regardless of portal of entry than landmark‐guided injection (96% vs. 78%).^9^, ^10^, ^11^ In addition, ultrasound devices are easily accessible, relatively inexpensive and have the advantage of real‐time visualisation of vascular and nervous structures and no risk of radiation.^12^ Despite these advantages, questions remain about translating higher accuracy rates of ultrasound‐guided injection into better clinical improvements.^13^ In addition, there is no systematic literature appraisal or meta‐analysis of this important aspect of clinical practice using patient‐reported outcomes.^11^

Therefore, our current systematic review was aimed to investigate the difference in pain (procedural pain and pain improvement), functional outcomes and patient satisfaction between ultrasound‐guided intra‐articular injection and landmark‐guided intra‐articular injection in randomised controlled clinical trials in knee, hip and hand osteoarthritis.

## Methods

### Selection criteria

The studies were eligible (i) if they were randomised clinical trials (RCTs) which compared ultrasound‐guided IA injection of any injectates (steroid/hyaluronic acid/PRP/ others) with landmark‐guided IA injection in knee, hip or hand OA and (ii) if the patient‐reported outcomes in terms of pain, function and satisfaction were reported. The studies were excluded (i) if they were observational studies, cohort studies, narrative reviews, commentary, editorials, conference proceedings, conference poster/abstract or (ii) if study participants were <18 years old or (iii) if the studies were conducted in conditions other than OA (e.g. tendinitis, bursitis) or (iv) if the study was not published in English. The study protocol was registered in the PROSPERO database with CRD42023454255.

### Information source and selection process

This study followed the 2009 PRISMA (Preferred Reporting Items for Systematic Reviews and Meta‐Analyses) guidelines and the PRISMA‐IPD statement. A comprehensive literature search was carried out using electronic databases PubMed, Medline and Embase from their respective inception to August 28, 2023. The databases were searched individually for all possible terms and combinations of terms to accommodate differences in their search engines. The keywords used in the searches were ‘Osteoarthritis’ OR ‘Osteoarthrosis’ AND ‘Ultrasound’ OR ‘Ultrasonography’ filtered by ‘human’ and ‘randomized controlled trial’. Two authors (WMO and MABS) screened studies for eligibility independently, and any disagreements were resolved through discussion with a third reviewer (DJH). Finally, a manual search of included bibliographies was conducted for any missed studies.

### Data extraction and quality assessment

One reviewer (WMO) conducted data extraction with a standardised excel template including: (i) study descriptions such as author, publication year, country, OA site, sample size, name and dosage of injectate used, follow‐up durations and any permission of rescue medication during the study; (ii) participants characteristics such as mean age and standard deviation (SD), sex, body mass index (BMI), diagnostic criteria used, (iii) technical features of ultrasound guidance such as description of methods, in‐plane or out‐of‐plane technique used, post‐injection instructions, any blinding to the procedures, confirmation of accuracy rates of the injectates, credential of the proceduralist and any presence of adverse effects from the procedure; (iv) patient‐reported outcomes measures such as two types of relevant pain outcomes on visual analogue scale or numeral rating scale [1. procedural pain (pain during the injection into joint) and 2. improvement in pain outcome using data at the longest follow‐up of the study)], functional outcomes for particular joint sites such as Western Ontario and McMaster Universities Arthritis Index (WOMAC)/Knee Injury and Osteoarthritis Outcome Score (KOSS)/Hip Injury and Osteoarthritis Outcome Score (HOSS)/Functional Index of Hand Osteoarthritis (FIHOA) and patient satisfaction as primary outcomes and accuracy rates of each methods as the secondary outcomes to plan a meta‐regression of accuracy rate for patient‐reported outcomes if there are at least 10 studies for each outcome.

One reviewer (WMO) performed quality assessment using the Jadad scale which awards points for 3 key methodological aspects of clinical trials: randomisation (up to two points are given), double blinding (up to two points are given) and accounting for withdrawals and drop‐outs (up to one point is given).^14^ Jadad scale ranges from 0 to 5 in total, with clinical trials scoring 3 or greater considered good quality trials. The same reviewer conducted a risk‐of‐bias assessment by using the Cochrane collaboration risk‐of‐bias assessment tool and assigned a value of ‘high’, ‘low’ or ‘unclear’ bias for each study using the following parameters: selection bias, performance bias, detection bias, attrition bias, reporting bias and other biases.^15^

## Statistical analysis

Results of selected studies in respective outcome measures were qualitatively summarised for each OA site or quantitatively pooled if two or more studies were available for each OA site. Separate meta‐analyses were performed for each type of patient‐reported outcome: pain, function and satisfaction. Pooled standardised mean difference with 95% confidence intervals (95% CI) and forest plots were performed, using the comprehensive meta‐analysis software version 3. We used random effects modelling to account for potential heterogeneity in study methodology and patient characteristics. The heterogeneity of studies was assessed using *I* ^2^, and publication bias using funnel plots for each outcome pooled. A P‐value less than 0.05 was deemed statistically significant, and all tests were two‐tailed. For the direction of effect size, negative convection was used for pain reduction, and positive convention was used for functional improvement. Standardised mean differences (SMDs) are interpreted as follows: 0.2 = small effect; 0.5 = moderate effect and 0.8 = large effect. If sufficient studies were available for each outcome (n ≥ 10), a meta‐regression of pain, function and patient satisfaction by accuracy rates of ultrasound guidance versus landmark guidance was planned to be conducted.

## Results

**Figure 1:**
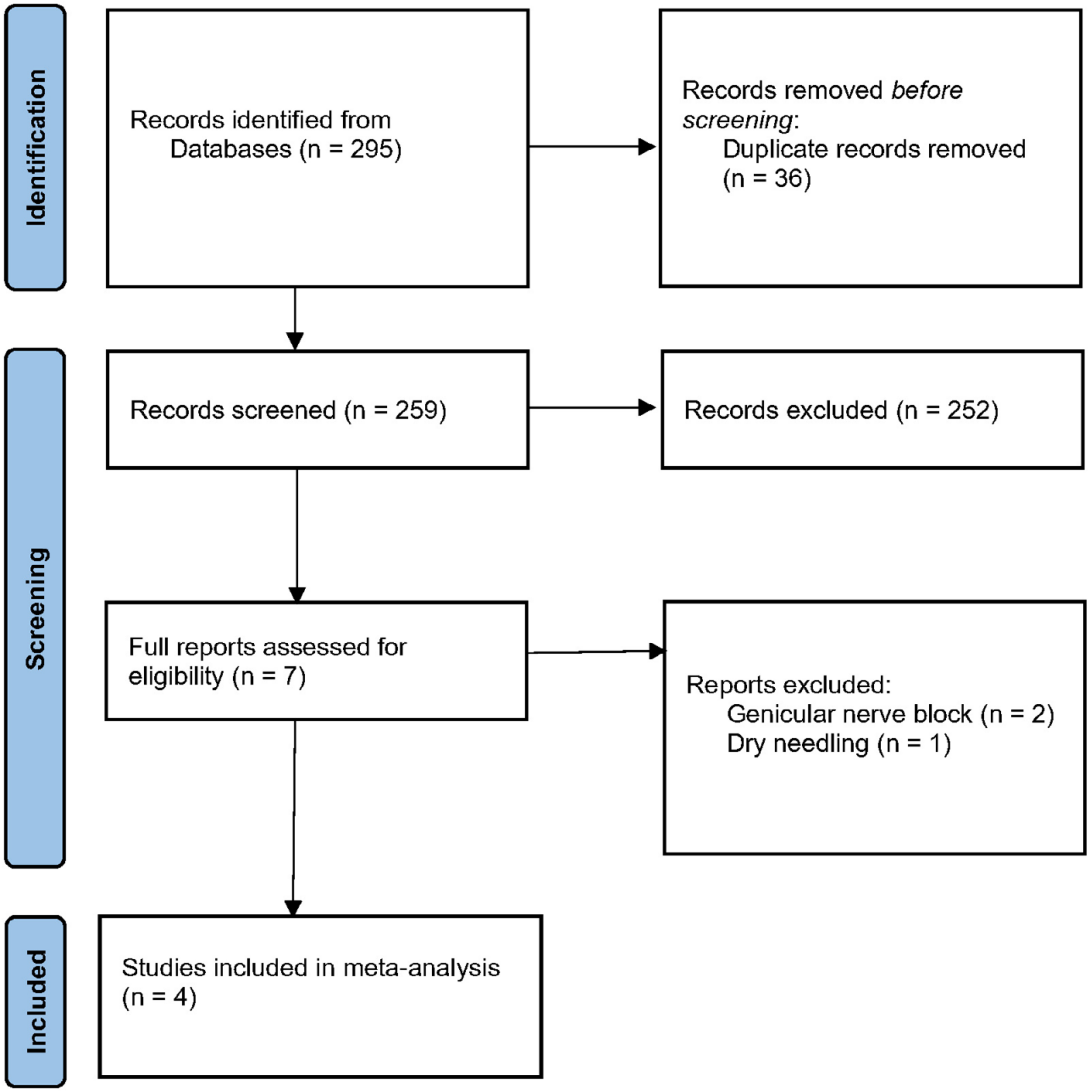
PRISMA flow diagram showing selection of studies.

Our search identified 295 records (73 PubMed, 47 Medline and 175 Embase), for which 36 duplicate and 252 unrelated studies were excluded after initial screening. Upon review of the remaining seven full‐text articles for knee OA, two were related to the genicular nerve around the knee [15, 16] and another related to dry needling.^16^ This resulted in four RCTs being included for meta‐analysis, totalling 338 participants included [18–21] as shown in the PRISMA flow diagram (Figure 1). No comparative studies of the hip or hand were identified.

The characteristics of the included studies are presented in Table 1. Three RCTs were from USA^17^, ^18^, ^19^ and one RCT from Iran.^20^ Three RCTs were done in knee OA^18^, ^19^, ^20^ and one study involved a mixed population with multi‐site OA and RA.^17^ Corticosteroid injectates were used in three studies,^17^, ^18^, ^19^ and hyaluronic acid injectate was used in one study.^20^

**Table 1:** Study characteristics of included studies.

Author	Country	OA site	Sample size	Age (mean ± SD), years	Female %	BMI (mean ± SD)	Diagnostic criteria	Injectate/Dosage	Follow‐ups	Rescues medication permitted	Adverse effects reported
Sibbitt *et al*., 2009	USA	Multi‐OA	100 RA 48 OA	Palpation: 55.5 ± 12.8 Sonographic: 51.7 ± 15.5	Palpation: 83.8%; Sonographic: 86.5%	Not reported	Any joint pain	IA triamcinolone acetonide	2 weeks	No	Unclear
Sibbitt *et al*., 2011	USA	Knee OA	92 OA	Palpation: 61.9 ± 9.9 Sonographic: 62.9 ± 9.9	Palpation: 87.0%; Sonographic: 87.0%	Palpation: 30.0 ± 7.2; Sonographic: 31.9 ± 6.1	Radiographic Brandt grades 1 to 3 (0–4)	80‐mg triamcinolone acetonide suspension	Procedural pain 2 weeks 6 months	Not reported	
Kianmehr *et al*., 2018	Iran	Knee OA	61 OA	61.52 ± 9.09	78.60%	27.66 ± 3.88	ACR and any KL grade	Hyaluronic acid	6 weeks 12 weeks	Not reported	
Sheth *et al*., 2021	USA	Knee OA	21 OA 11RA 5 crystal arthropathy	Palpation: 58.3 ± 9.6 Sonographic: 60.9 ± 10.4	Palpation: 66.7%; Sonographic: 73.7%	Not reported	Any knee pain	40 mg of depomedrol and 3 cc of 1% lidocaine	Procedural pain; 4 weeks	Not reported	

ACR, American College of Rheumatology; BMI, body mass index; IA, intra‐articular; KL, Kellgren and Lawrence; OA, osteoarthritis; RA, rheumatoid arthritis; SD, standard deviation; USA, United States of America.

Regarding technical aspects of ultrasound (Table 2 **)**, all studies reported sufficient description of ultrasound‐guided or landmarked‐guided procedures but there was limited information related to blinding of joint injection techniques, post‐injection instructions and accuracy confirmation. No accuracy rates were reported in the included studies.

**Table 2:** Technical characteristics of included studies.

Author	Description of ultrasound approach	In plane/Out of plane	Blinding to imaging	Post‐injection instruction	Accuracy confirmation	Credential of proceduralist
Sibbitt *et al*., 2009	Yes	In plane	Unclear	No	No	Not mentioned
Sibbitt *et al*., 2011	Yes	In plane	Not reported	Yes	No	Fellows‐in‐training proceduralists
Kianmehr *et al*., 2018	Yes	Not reported	Yes	Not reported	No	Rheumatologist
Sheth *et al*., 2021	Yes	In plane	No blinding	No	No	Not mentioned

### Quality assessment

Only one study was deemed as high quality^20^ on the Jadad score and the remaining three studies lost points due to a lack of double blinding^17^, ^18^, ^19^ (Table 3). Two studies did not report the number and the reasons for withdrawal.^19^, ^20^

**Table 3:** Jadad scores of included studies.

Study	Was the study described as randomised?	Was the randomisation scheme described and appropriate?	Was the study described as double blind?	Was the method of double blinding appropriate?	Was there a description of dropouts and withdrawals?	Total
Sibbitt *et al*., 2009	1	0	0	0	1	2
Sibbitt *et al*., 2011	1	0	0	0	1	2
Kianmehr *et al*., 2018	1	1	1	1	0	4
Sheth *et al*., 2021	1	0	0	0	0	1

A study receives a score of 1 for ‘yes’ and 0 for ‘no’. The score ranges from 0 to 5.

### Risk of bias

**Figure 2:**
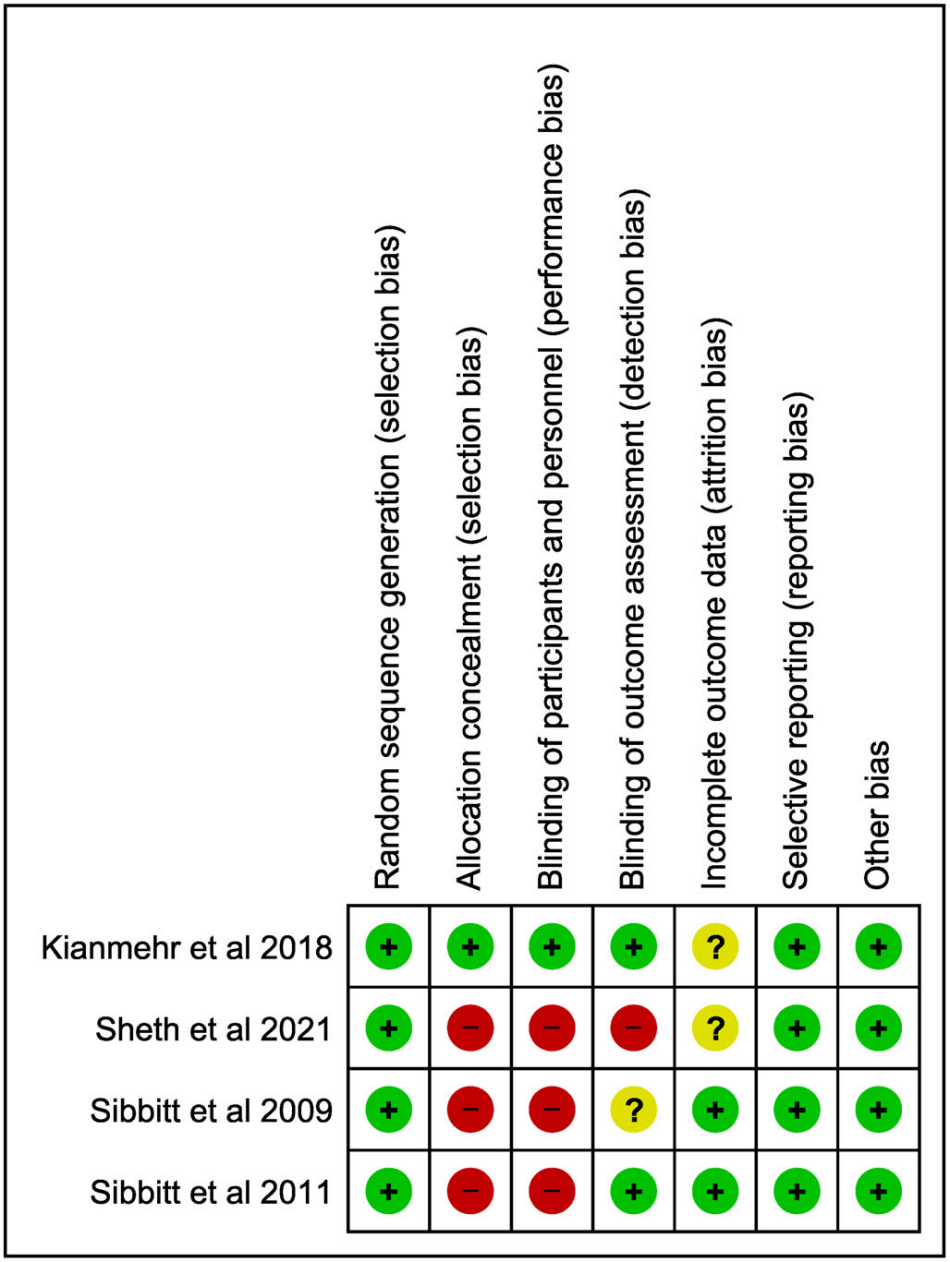
Risk of bias of four included studies.

**Figure 3:**
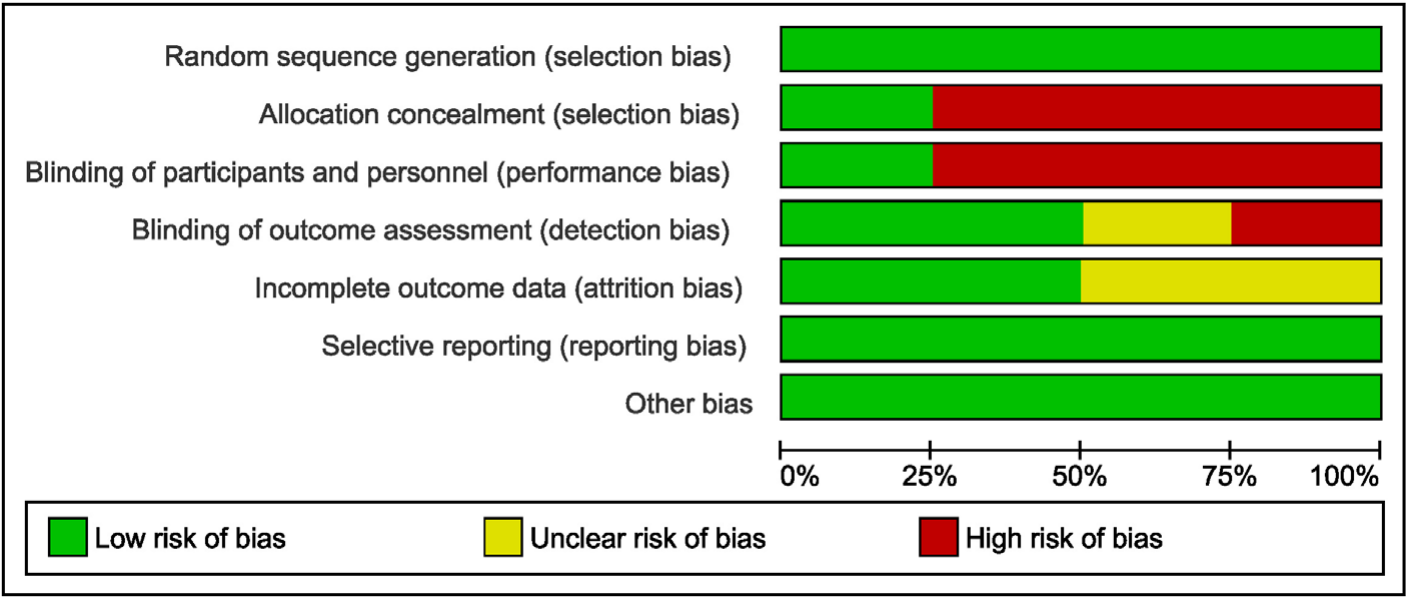
Summary of risks of bias.

Risk of bias for each study is illustrated in Figure 2. One out of the four studies (33.3%) was considered ‘low risk’ of bias,^20^ whereas the remaining three studies were graded at ‘some concerns’ or had ‘high risk’ of bias^17^, ^18^, ^19^ based on the Cochrane risk‐of‐bias tool. A summary of the risks of bias across the included studies is presented in Figure 3. Allocation concealment and blinding of participants and personnel were sources of bias that affected the majority of studies (75%).

### Procedural pain

**Figure 4:**
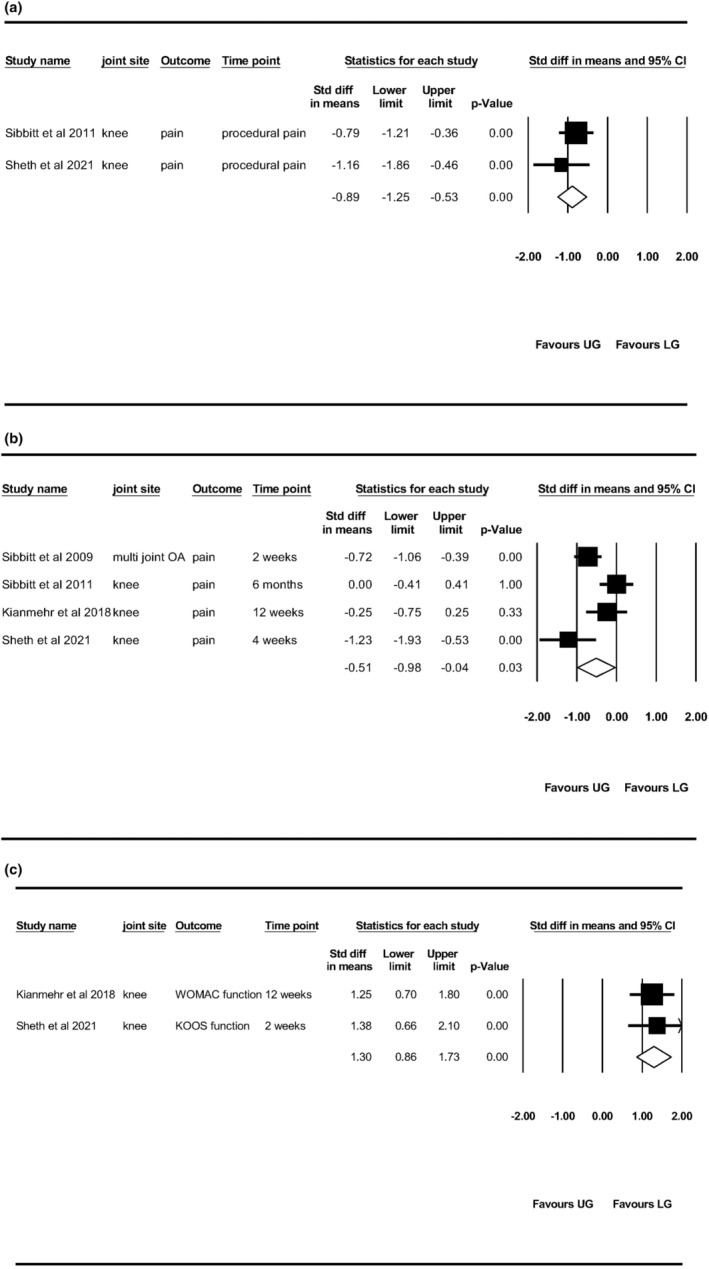
Pooled results of included studies for (a) procedural pain, (b) pain reduction and (c) function. Abbreviation: KOOS, Knee Injury and Osteoarthritis Outcome Score; LG, landmark guided; OA, osteoarthritis; UG, ultrasound guided, WOMAC, Western Ontario and McMaster Universities Arthritis Index.

Ultrasound‐guided IA injection demonstrated a significant large effect size in reducing pain during the injection procedure compared with landmark‐guided IA injection [−0.89 (95% CI ‐1.25, −0.53)] with no heterogeneity (*I* ^2^ = 0%), in pooling two RCTs reporting this outcome measures^18^, ^19^ (Figure 4a).

### Pain reduction in follow‐ups

The longest follow‐up of four RCTs is variable with 2 weeks,^17^ 4 weeks,^19^ 12 weeks^20^ and 6 months^18^ respectively. Combining the data demonstrated a significant moderate effect size in pain reduction for ultrasound‐guided IA injection in comparison with landmark‐guided IA injection [−0.51 (95% CI −0.98, −0.04)] with small heterogeneity (*I* ^2^ = 13%; Figure 4b **)**.

### Functional improvement

Two RCTs reported functional improvement at 2 weeks^19^ and 12 weeks,^20^ respectively, with the pooled results indicating a significant large improvement in function for the ultrasound‐guided IA injection compared with landmark‐guided IA injection [1.30 (95% CI 0.86, 1.73)]. No heterogeneity was identified (*I* ^2^ = 0%; Figure 4c **)**.

### Patient satisfaction

Only one RCT studied patient satisfaction as the outcome for comparing the two methods, reporting that ultrasound guidance provided higher patient satisfaction immediately after the procedure (4.9 vs. 4.1, P = 0.01) and after 4–6 weeks of follow‐up (4.5 vs. 3.4 ± 1.6, P = 0.03) on a 5‐point Likert scale (the higher, the more satisfied).

## Discussion

This is the first meta‐analysis examining the symptomatic benefits of ultrasound‐guided IA injection compared to landmark‐guided IA injection in osteoarthritis. Our study revealed that ultrasound‐guided IA injection provided significant improvements in patient‐reported outcomes such as pain, function and patient satisfaction compared with landmark‐guided IA injection.

Intra‐articular injections performed without imaging guidance are generally quite inaccurate (78%), even in large joints such as the knee.^11^ As therapeutic effectiveness may be contingent upon the accurate administration of injectate, using anatomic landmarks to guide injection may be inadequate for obtaining the maximal clinical benefits.^9^ On the other hand, ultrasound guidance of knee injections resulted in better accuracy than anatomical guidance (96% vs. 78%).^10^ However, previous reviews focus only on the accuracy rates of the two methods and did not look at the patient‐reported outcomes to inform any difference in clinical benefits.^10^, ^11^, ^21^

In 2016 EULAR (European League against Rheumatic Disease) guideline related to OA management, the option of imaging‐guided injections was left for specific situations, identified by the experts, due to a paucity of data in clinical outcomes despite documented evidence of higher accuracy rate.^22^ With the emerging data since publications of the guideline, it is plausible that ultrasound‐guided IA injection provided greater pain relief and functional improvement at least in the short‐term up to 6 months, supported by our current review and meta‐analysis. More complete removal of synovial fluid effusions from the OA knee has been shown to improve OA injection outcomes^23^, ^24^, ^25^; it is likely that ultrasound permitted more complete arthrocentesis prior to injecting the IA therapy, thus improving the outcomes. In addition, newer therapies for osteoarthritis such as joint‐specific cellular therapy may have to be delivered exactly and thus ultrasound guidance may be required for certain of these therapies.^26^

Similar to this meta‐analysis, a 2023 systematic review in shoulder pain comparing the two techniques demonstrated a significant reduction in pain [SMD −0.48, 95% CI (−0.79, −0.17)] and functional improvement [SMD 0.35, 95% CI (0.05, 0.65)], favouring the ultrasound‐guided injection.^27^ In a 2018 narrative review related to ultrasound‐guided injection in sports medicine, superior clinical outcomes were noted for the glenohumeral joint, the subacromial space, the biceps tendon sheath and the joints of the hand and wrist, the knee, ankle and foot.^28^

In addition to the difference in clinical outcomes between the two techniques, our meta‐analysis also demonstrated the significant difference in procedure‐related pain which is important for patient's acceptability. This can be explained by better control of the needle, resulting in less intra‐articular bleeding, less tissue trauma, reduced pain and less bruising.^29^ Moreover, ultrasound may also decrease pain by providing distraction and counterirritation through the pressure of the probe, the coolness of the ultrasound gel, and movement upon the skin. Ultrasound guidance may be important to avoid procedural complications related to inadvertent puncture of blood vessels and nerve structures.^9^, ^30^

As a note, anatomic landmark corticosteroid injections have shown some degree of efficacy even when they are not delivered to the joint space.^6^ This may be due to the fact that once corticosteroid crystals are injected in the soft tissues in the proximity to an intended injection target, the corticosteroid can move via lymphatics or systemically, which may explain why exact accuracy is not always necessary.^31^

Our review has some limitations. Only a few studies examined the functional improvements as an outcome and only one evaluated patient satisfaction. Moreover, these studies did not all include important standard metrics of outcomes for OA trials including the WOMAC, Oxford knee score and others^32^, ^33^; thus, the results produced from the meta‐analysis would be considered preliminary. There were some differences in patient characteristics and durations of follow‐ups although heterogeneity was insignificant in our analyses (*I* ^2^ = 0–13%). Long‐term data of more than 6 months are not available, requiring further studies.

## Conclusion

Our meta‐analysis demonstrated a significant improvement in patient‐reported outcome measures such as pain, function and patient satisfaction with ultrasound‐guided IA injection compared with landmark‐guided IA injection. Further studies evaluating the cost‐effectiveness of ultrasound‐guided IA injection compared with landmark‐guided IA injection are needed for informing clinical practice.

## Funding

This research received no external funding.

## Conflict of interest

DJH is the co‐director of the Sydney Musculoskeletal Health Flagship. In addition, DJH is the editor of the osteoarthritis section for UpToDate, co‐editor in Chief of Osteoarthritis and Cartilage and board member of Osteoarthritis Research Society International. DJH provides consulting advice on scientific advisory boards for Pfizer, Lilly, TLCBio, Novartis, Tissuegene and Biobone. All other authors declare no conflict of interest for this article.

## Author contributions

WMO and DJH were involved in the conceptualisation and study design; WMO and MABS in the selection of papers; WMO, JL, AS, KF and DJH were involved in analysis and interpretations. WMO drafted the first manuscript and all authors read and agreed to the final version of the manuscript.
